# One-step radiosynthesis of the MCTs imaging agent [^18^F]FACH by aliphatic ^18^F-labelling of a methylsulfonate precursor containing an unprotected carboxylic acid group

**DOI:** 10.1038/s41598-019-55354-w

**Published:** 2019-12-11

**Authors:** Masoud Sadeghzadeh, Rareş-Petru Moldovan, Rodrigo Teodoro, Peter Brust, Barbara Wenzel

**Affiliations:** Helmholtz-Zentrum Dresden-Rossendorf, Institute of Radiopharmaceutical Cancer Research, Research Site Leipzig, Permoserstrasse 15, 04318 Leipzig, Germany

**Keywords:** Nuclear chemistry, Analytical chemistry

## Abstract

Monocarboxylate transporters 1 and 4 (MCT1 and MCT4) are involved in tumour development and progression. Their level of expression is particularly upregulated in glycolytic cancer cells and accordingly MCTs are considered as promising drug targets for treatment of a variety of human cancers. The non-invasive imaging of these transporters in cancer patients via positron emission tomography (PET) is regarded to be valuable for the monitoring of therapeutic effects of MCT inhibitors. Recently, we developed the first ^18^F-radiolabelled MCT1/MCT4 inhibitor [^18^F]FACH and reported on a two-step one-pot radiosynthesis procedure. We herein describe now a unique one-step radiosynthesis of this radiotracer which is based on the approach of using a methylsulfonate (mesylate) precursor bearing an unprotected carboxylic acid function. With the new procedure unexpected high radiochemical yields of 43 ± 8% at the end of the radiosynthesis could be obtained in a strongly reduced total synthesis time. Moreover, the radiosynthesis was successfully transferred to a TRACERlab FX2 N synthesis module ready for future preclinical applications of [^18^F]FACH.

## Introduction

Metabolic reprogramming is one of the two emerging hallmarks of cancer postulated by Hanahan and Weinberg in 2011^1^. First observed by Warburg^2^, tumour cells primarily produce energy via switching from mitochondrial oxidative phosphorylation (MOP) to aerobic glycolysis even in the presence of oxygen^3,4^. Aerobic glycolysis is less energy efficient than MOP, but it appears to confer advantages for rapidly proliferating cells through the formation of metabolites like e.g. lactate, which can be used as a prominent substrate that fuels the metabolism of oxidative tumour cells^2^. To transport these metabolites across plasma membranes and avoiding intracellular and/or extracellular acidosis, glycolytic cancer cells upregulate H^+^-linked membrane proteins such as monocarboxylate transporters (MCTs) which belong to the solute carrier 16 (SLC16) gene family that contains 14 members in humans and mice^5^. Among them, MCT1 and MCT4 are the ones which are most widely expressed in several cancers including breast, prostate, colorectal, and lung tumours as well as gliomas^6–10^. It has been reported that these MCTs play an important role in tumour proliferation and malignancy. Accordingly, they have been proposed as therapeutic targets for various cancer types and in particular for high-grade brain tumours, whose energy metabolism presumably relies on the Warburg effect^9,10^. In this regard, previous studies have also demonstrated that MCT1 RNA interference (RNAi) causes cell death in glioma cell lines^11^.

Although, MCTs were proposed as promising biomarker candidates, their relevance *in vivo* has not been well assessed so far by using non-invasive imaging techniques like PET. Therefore, there is a need for the development of potent inhibitors of MCTs radiolabelled with a suitable short-lived PET radionuclide such as ^18^F-fluorine (*t*_1/2_ = 109.7 min) to evaluate them regarding their potential for cancer diagnosis and therapy monitoring.

**Figure 1:**
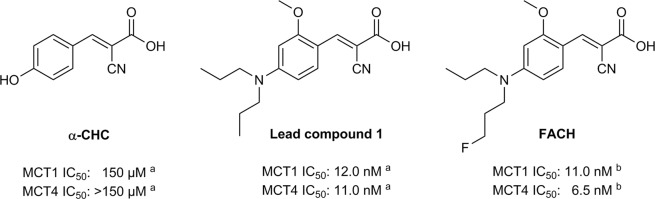
The potent MCT1 inhibitor **1** as lead compound and its corresponding fluorinated analogue FACH on the basis of the *α*-CHC core structure. (**a**) Data published in^15^. (**b**) Data published in^14^.

To develop an ^18^F-labelled radiotracer suitable for imaging of MCT1, we selected 1 as lead compound (Fig. 1) belonging to the class of *α*-cyano-4-hydroxycinnamic acids (*α*-CHC)^12^ as it was described with high MCT1 inhibitory activity (IC_50_: 12.0 nM)^13^. Compound 1 was a result of a comprehensive structure-activity-relationship study based on a series of *α*-CHC derivatives, discovering important structural requirements for high MCT1 inhibition: i) 2-cyanoacrylic acid moiety, ii) *p*-*N*-dialkyl or -diaryl function instead of the OH group and iii) *o*-methoxy group on the phenyl ring^13^. Therefore, replacing one of the *N*-substituted propyl groups by a 1-fluoropropyl function did not significantly change the inhibitory potency and resulted in the development of the recently published new potent MCT1 inhibitor FACH (Fig. 1)^14^. Current studies have revealed that both, 1 and FACH, also possess high inhibition toward MCT4 (IC_50_: 11.0 nM for 1 and 6.5 nM for FACH)^14,15^, making these compounds even more attractive as drug and imaging probes. Accordingly, FACH was radiofluorinated by our group in order to develop the first ^18^F-labelled MCTs inhibitor for PET imaging^14^.

**Figure 2:**
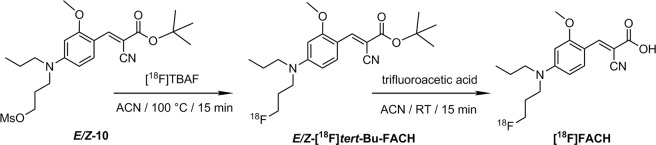
Previously reported two-step one-pot radiosynthesis of [^18^F]FACH^14^.

The radiosynthesis of [^18^F]FACH was developed as a two-step one-pot procedure using a precursor containing a methylsulfonate (mesylate, OMs) leaving group (10) and a *tert*-butyl protected carboxylic acid function (Fig. 2)^14^. Nucleophilic aliphatic substitution of the mesylate group by [^18^F]fluoride in the first step yielded the intermediate [^18^F]*tert*-Bu-FACH which was deprotected under acidic conditions in the second step. The radiolabelling was investigated using the common K[^18^F]F-K_2.2.2_-carbonate system and tetra-*n*-butylammonium [^18^F]fluoride ([^18^F]TBAF) as fluorination agents. While with the K[^18^F]F-K_2.2.2_-carbonate system a considerable amount of a radioactive byproduct was formed depending on the amount of base, [^18^F]TBAF proved to be beneficial regarding radiochemical yield and reproducibility. However, several problems emerged when searching for a suitable deprotection system, of which the best was found to be trifluoroacetic acid at room temperature followed by neutralization with triethylamine and purification of [^18^F]FACH by semi-preparative HPLC. Finally, with this manual synthesis the radiotracer could be obtained with a radiochemical yield (RCY) of 39.6 ± 8.3% in a total radiosynthesis time of about 160 min.

Encouraged by the very promising first preclinical results obtained from PET studies with [^18^F]FACH in mice^16^, we reconsidered our two-step radiosynthesis procedure. With the goal to reduce the synthesis time and to simplify the process for translation into an automated synthesis module for future preclinical studies, we envisaged a one-step radiosynthesis of [^18^F]FACH using an unprotected precursor.

According to the general opinion, aliphatic and aromatic nucleophilic substitution reactions with [^18^F]fluoride are challenging when the molecules contain reactive functionalities such as amino or carboxylic acid groups^17,18^. Usually, the corresponding precursors have to be derivatised with appropriate protecting groups, since [^18^F]fluoride can easily react with acidic protons to form hydrogen fluoride. This is well reflected by numerous publications describing the nucleophilic ^18^F-labelling of compounds at which either the reactive functionalities were protected or a multi-step procedure with ^18^F-labelled prosthetic groups was used^17,19^. A well known example is the amino acid *O*-[^18^F]fluoroethyl-L-tyrosine ([^18^F]FET), which was first synthesised by a two-step procedure using [^18^F]fluoroethyl tosylate as prosthetic group coupled to tyrosine^20^, and later on optimised to a one-pot procedure via using a protected tyrosine derivative and direct ^18^F-labelling^21^. Also radiotracers containing solely a carboxylic function such as the ^18^F-labelled MCT substrate pyruvate ([^18^F]fluoropyruvate)^22^, [^18^F]fluoroacetate ([^18^F]FA)^23^ or [^18^F]fluorpropionic acid ([^18^F]FPA)^24^ were synthesised by using the corresponding ester derivatives as precursor compounds. Only very few examples are reported for ^18^F-labellings without protection of reactive functionalities such as recently the one-step radiosynthesis of [^18^F]PSMA-1007^25^. In this procedure a peptidomimetic substance containing several carboxylic acid groups was directly radiofluorinated by nucleophilic heteroaromatic substitution of a trimethylammonium leaving group with [^18^F]fluoride. Compared to the formerly described two-step radiosynthesis of [^18^F]PSMA-1007, considerably higher radiochemical yields could be obtained with the one-step procedure^25^. However, to the best of our knowledge, aliphatic nucleophilic radiofluorination of compounds with unprotected carboxylic acid functionalities has not been described so far.

We herein report on the development of a one-step radiosynthesis of the novel MCT1/MCT4 targeting radiotracer [^18^F]FACH on the basis of a nucleophilic aliphatic ^18^F-labelling procedure using a mesylated precursor with an unprotected carboxylic acid function. Furthermore, we describe the successful translation of the new procedure to an automated radiosynthesis module (GE Tracerlab FX2 N).

## Results and Discussion

### Synthesis of the unprotected precursor 11

**Figure 3:**
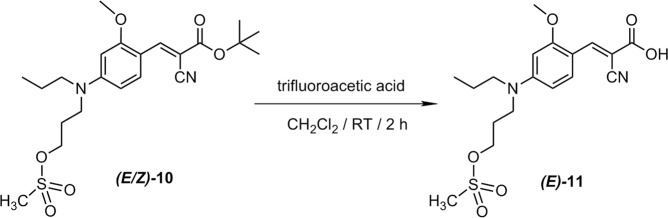
Synthesis of the unprotected mesylated precursor ***(E)***-**11**.

Based on the good results obtained for the aliphatic nucleophilic substitution of a mesylate group by [^18^F]fluoride in the two-step radiosynthesis of [^18^F]FACH^14^, we decided to maintain this leaving group. The synthesis of the desired unprotected precursor **11** was performed starting from the previously reported *tert*-butyl protected precursor **10**^14^. Finally, **11** was achieved by removal of the *tert*-butyl group of **10** under acidic conditions in nearly quantitative yield (Fig. 3). According to HPLC and NMR analysis, this reaction step led to the solely formation of the *E* isomer of **11**, while **10** was applied as a mixture of *E* and *Z* isomer in a ratio of 4:1. Compound **11** was stored at −30 °C and remained stable over a period of at least several months.

### Manual one-step radiosynthesis of [^18^F]FACH

Based on our former experiments with the *tert*-butyl protected precursor **10**^14^, we first selected [^18^F]TBAF as fluorination agent and acetonitrile (ACN) and *tert*-butanol as most promising solvents. HPLC analysis of samples taken from the crude reaction mixture revealed the generation of [^18^F]FACH in ACN with radiochemical yields of 54 ± 7% (n = 4) after 15 minutes reaction time at 100 °C (thermal heating, Table 1). An increase of the reaction time did not result in higher radiochemical yields. A single experiment using 1.0 and 2.0 mg of the precursor (**11**) under the same reaction conditions indicated the independence of this labelling process on the precursor amount. Therefore, the labelling experiments could be performed with only 1.0 mg of precursor. Also with *tert*-butanol as reaction medium the formation of [^18^F]FACH could be observed, however, to a much less extent (RCY: ~10%). When the precursor was applied as its sodium salt **11**-Na, the radiochemical yield also decreased to 9 ± 2% (n = 2).

**Figure 4:**
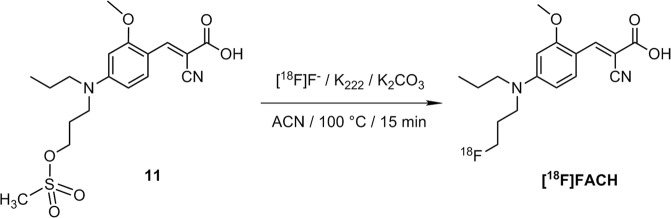
One-step radiosynthesis of [^18^F]FACH using the unprotected precursor 11.

**Figure 5:**
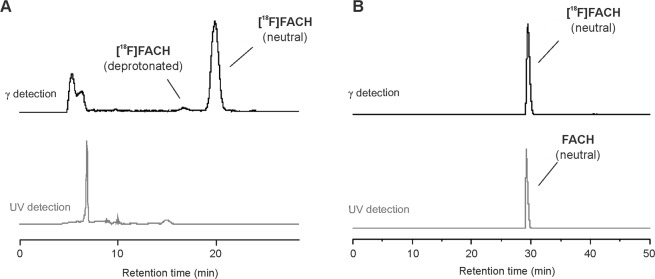
(**A**) Representative semi-preparative radio- and UV-HPLC chromatograms of [^18^F]FACH (conditions: Reprosil-Pur C18-AQ, 250 × 10 mm, 50% ACN/aq_._ 20 mM NH_4_HCO_2_, 3.5 mL/min). (**B**) Analytical radio- and UV-HPLC chromatograms of the final product of [^18^F]FACH spiked with the nonradioactive reference FACH (conditions: Reprosil-Pur C18-AQ, 250 × 4.6 mm, gradient with an eluent mixture of ACN/aq. 20 mM NH_4_OAc, 1.0 mL/min).

After these surprising results we were curious to additionally investigate the K[^18^F]F-K_2.2.2_-carbonate system for the one-step radiosynthesis of [^18^F]FACH, as during the two-step radiosynthesis a considerable amount of a single radioactive by-product was formed depending on the amount of base. However, using 11.0 mg (29.0 μmol) of K_2.2.2_ and 1.8 mg (13 μmol) of K_2_CO_3_ in ACN, an increase of the RCY up to 66 ± 12% (n = 8) was observed for the new labelling process of [^18^F]FACH at 100 °C with an optimal reaction time of 15 minutes (Fig. 4). According to radio-HPLC analysis, also a single radioactive by-product was formed in the reaction mixture, however, accounting for less than 5% of the total activity (Fig. S4 in Supplementary Information). Compared to the results obtained with the *tert*-butyl protected precursor **10** via the two-step procedure, it seems that the radiolabelling with the K[^18^F]F-K_2.2.2_-carbonate system and the unprotected precursor is unexpectedly more robust. Therefore, we went further with this system for completion of the one-step radiosynthesis of [^18^F]FACH. The isolation of the radiotracer was performed by using semi-preparative RP-HPLC. The product was collected at a retention time of about 20 min (A in Fig. 5), afterwards purified using solid phase extraction (SPE) on an RP cartridge, and formulated in sterile isotonic saline containing 10% of EtOH. Analytical radio- and UV-HPLC of the final product with co-elution of the non-labelled reference compound confirmed the identity of [^18^F]FACH (B in Fig. 5). Finally, the radiotracer was obtained with a radiochemical purity of ≥ 98%, radiochemical yields of 43 ± 8% (n = 8, decay corrected to the end of bombardment, EOB), and molar activities in the range of 50–120 GBq/µmol (at the end of synthesis, EOS) using starting activities of 1–3 GBq.

The stability of the radiotracer was investigated by incubation of [^18^F]FACH in *n*-octanol, saline and phosphate buffered saline (PBS) at 40 °C. The radiotracer proved to be stable in these media, and no defluorination or degradation was observed within 60 min of incubation time. To estimate the lipophilicity of [^18^F]FACH, the logD value was determined by the shake flask method using *n*-octanol and PBS (pH 7.4) as partition system. With a logD_7.4_ value of 0.42 ± 0.02 (n = 4) the radiotracer is rather hydrophilic.

As previously described in detail^14^, two species of [^18^F]FACH could be observed in radio-HPLC chromatograms depending on the pH value and solvent composition of the samples. Thus, at a sample pH value lower than 6 mainly the neutral form is present. In order to achieve an efficient semi-preparative isolation of the radiotracer during the radiosynthesis, the reaction mixture was therefore diluted with aqueous ammonium formate (adjusted to pH 4.0 with formic acid) before loading on the column. As can be seen in Fig. 5A, mainly the neutral form of [^18^F]FACH was available.

### Automated radiosynthesis of [^18^F]FACH

Based on the results of the manual experiments, the radiosynthesis of [^18^F]FACH was transferred to an automated procedure using a TRACERlab FX2 N synthesis module (GE Healthcare). The setup of the module is described in the experimental part. Briefly, after trapping and elution of [^18^F]fluoride from an anion exchange cartridge, the labelling reaction of the azeotropically dried [^18^F]F^−^/K_2.2.2._/K_2_CO_3_ complex with the unprotected precursor (**11**) was performed in ACN for 12 min at 100 °C. For isolation of [^18^F]FACH, the crude reaction mixture was diluted with a mixture of aqueous ammonium format (pH 4.0) and acetonitrile and then directly applied to the implemented semi-preparative HPLC system. The radiotracer fraction was collected at a retention time of about 20 min and purified by solid phase extraction using a C18-cartridge. The obtained radiotracer solution was transferred out of the hot cell, concentrated under argon stream and formulated in sterile isotonic saline containing 10% of ethanol. The entire process lasts about 80 min. Activity balance showed that less than 2% of the activity was lost in the anion exchange cartridge and during SPE. Finally, [^18^F]FACH could be produced with a radiochemical purity of ≥ 98%, an RCY of 34.3 ± 4.8% (n = 5) and molar activities between 65–330 GBq/µmol (n = 5) at starting activities of 2–6 GBq.

Notably, at the beginning of the experiments in the synthesis module a high variability of the radiochemical yields (labelling step) was observed ranging from 5 to 80%, which was not found during the manual experiments. We discovered that already traces of water seemed to impede a successful labelling of [^18^F]FACH, a phenomenon which we did not observe with other produced radiotracers before. Accordingly, a modification of the complex drying step (details in Materials and Methods) as well as the cleaning procedure of the device was needed and resulted in a good reproducibility of the radiochemical yields.

In summary, a new one-step radiosynthesis of the MCT1/MCT4 targeting radiotracer [^18^F]FACH was developed. Using the unique approach of aliphatic nucleophilic radiofluorination of a precursor bearing an unprotected carboxylic acid function, remarkably high radiochemical yields of 66 ± 12% for the ^18^F-labelling step could be obtained. The total radiosynthesis time could be strongly reduced to about 80 minutes compared to 160 minutes needed for the former two-step procedure. Moreover, the new procedure was successfully transferred to the TRACERlab FX2 N synthesis module, necessary for future preclinical and clinical applications of the radiotracer.

## Materials and Methods

### Organic chemistry

#### General methods

All chemicals and reagents were purchased from commercial sources and used without further purification. For thin-layer chromatography (TLC), Silica gel 60 F254 plates (Merck KGaA, Darmstadt, Germany) were used. Room temperature was 21 °C. For mass spectrometry (MS), Finnigan MAT GCQ (Thermo Finnigan MAT GmbH, Bremen, Germany) was used. ^1^H and ^13^C spectra were recorded on VARIAN “MERCURY plus” (300 MHz for ^1^H NMR, 75 MHz for ^13^C NMR) and VARIAN “MERCURY plus” and BRUKER DRX-400 (400 MHz for ^1^H NMR, 100 MHz for ^13^C NMR, 377 MHz); *δ* in ppm related to tetramethylsilane; coupling constants (*J*) are given with 0.1 Hz resolution. Multiplicities of NMR signals are indicated as follows: s (singlet), d (doublet), t (triplet), m (multiplet), dd (doublet of doublets). ESI/Ion trap mass spectra (LRMS) were recorded with a Bruker Esquire 3000 plus instrument (Billerica, MA, USA). High resolution mass spectra were recorded on a FT-ICR APEX II spectrometer (Bruker Daltonics; Bruker Corporation, Billerica, MA, USA) using electrospray ionization (ESI) in positive ion mode^14,26^.

#### Organic syntheses

About 2–3 mg of the nonradioactive compound FACH were taken from a stock recently synthesised by our group^14^ and used as reference for analytical TLC and HPLC experiments. Also for the synthesis of the unprotected precursor 11 the stated amount of *tert*-butyl protected compound 10 was taken from a stock recently synthesised^14^.

(*E*)−2-Cyano-3-(2-methoxy-4-((3-((methylsulfonyl)oxy)propyl)(propyl)amino)phenyl)acrylic acid (**11**).

Trifluoroacetic acid (200 µL) was added to a solution of *tert*-butyl-(*E*)-2-cyano-3-(2-methoxy-4-((3-((methylsulfonyl)oxy)propyl)(propyl)amino)phenyl)acrylate **10** (20 mg, 0.044 mmol) in 200 µL CH_2_Cl_2_ and stirred at room temperature for 2 hours. After completion of the reaction, as judged by TLC (silica, ethylacetate/*n*-hexane, 1/1), the solvent was removed under reduced pressure and the residue was washed once with 2 mL diethyl ether to remove trace impurities. The desired compound **11** was obtained as yellow solid with a yield of >95% and used without further purification (NMR spectra and HPLC chromatogram are available in Supplementary Information, Figs. S1–S3).

^1^H NMR (400 MHz, DMSO-*d*_6_) *δ* 13.06 (s, 1H), 8.43 (s, 1H), 8.22 (d, *J* = 9.2 Hz, 1H), 6.55 (dd, *J* = 9.4, 2.3 Hz, 1H), 6.22 (d, *J* = 2.3 Hz, 1H), 4.31 (t, *J* = 6.0 Hz, 2H), 3.90 (s, 3H), 3.57 (t, *J* = 7.5 Hz, 2H), 3.42 (t, *J* = 7.7 Hz, 2H), 3.22 (s, 3H), 2.00 (q, *J* = 5.7, 5.3 Hz, 2H), 1.61 (m, 2H), 0.92 (t, *J* = 7.3 Hz, 3H). ^13^C NMR (101 MHz, DMSO-*d*_6_) *δ* 165.79, 162.14, 154.39, 146.71, 130.37, 118.93, 108.32, 105.98, 93.83, 91.76, 68.73, 56.24, 52.23, 47.09, 37.05, 27.02, 20.68, 11.53. HRFT-MS (ESI+): *m*/*z* = 397.1416 (calcd. 397.1433 for C_18_H_25_N_2_O_6_S^+^ [M + H]^+^).

For conversion of **11** into its sodium salt **11-**Na (sodium (*E*)-2-cyano-3-(2-methoxy-4-((3-((methylsulfonyl)oxy)propyl)(propyl)amino)phenyl)acrylate), an aqueous NaHCO_3_ solution (0.5 mL H_2_O, 2.7 mg, 0.032 mmol NaHCO_3_) was added to **11** (10 mg, 0.029 mmol) dissolved in 5 mL MeOH. The mixture was stirred at room temperature until a clear solution was formed (2 hours). Evaporation of the solvent gave **11**-Na as yellow solid, which was used for radiolabelling without further purification.

### Radiochemistry

#### General

No-carrier-added [^18^F]fluoride was produced via the [^18^O(p,n)^18^F] nuclear reaction by irradiation of an [^18^O]H_2_O target (Hyox 18 enriched water, Rotem Industries Ltd, Israel) on a Cyclone 18/9 (iba RadioPharma Solutions, Belgium) with fixed energy proton beam using Nirta [^18^F]fluoride XL target.

Radio thin layer chromatography (radio-TLC) was performed on silica gel (Polygram® SIL G/UV_254_) pre-coated plates with a mixture of CH_2_Cl_2_/MeOH 4/1 (v/v) as eluent. The plates were exposed to storage phosphor screens (BAS IP MS 2025 E, GE Healthcare Europe GmbH, Freiburg, Germany) and recorded using the Amersham Typhoon RGB Biomolecular Imager (GE Healthcare Life Sciences). Images were quantified with the ImageQuant TL8.1 software (GE Healthcare Life Sciences).

Analytical chromatographic separations were performed on a JASCO LC-2000 system, incorporating a PU-2080*Plus* pump, AS-2055*Plus* auto injector (100 μL sample loop), and a UV-2070*Plus* detector coupled with a gamma radioactivity HPLC flow detector (Gabi Star, raytest Isotopenmessgeräte GmbH). Data analysis was performed with the Galaxy chromatography software (Agilent Technologies) using the chromatograms obtained at 254 and 400 nm. A Reprosil-Pur C18-AQ column (250 × 4.6 mm; 5 µm; Dr. Maisch HPLC GmbH; Germany) with ACN/aq. 20 mM NH_4_OAc (pH 6.8) as eluent mixture and a flow of 1.0 mL/min was used (gradient: eluent A 10% ACN/aq. 20 mM NH_4_OAc; eluent B 90% ACN/aq. 20 mM NH_4_OAc; 0–5 min 100% A, 5–35 min up to 55% B, 35–36 min up to 100% B, 36–40 min 100% B, 40–41 min up to 100% A, 41–50 min 100% A); isocratic: 34% ACN/aq. 20 mM NH_4_OAc.

Semi-preparative HPLC separations were performed by using the HPLC system implemented in the TRACERlab FX2 N synthesizer (GE Healthcare, USA). A Reprosil-Pur C18-AQ column (250 × 10 mm; 10 µm; Dr. Maisch HPLC GmbH; Germany) and a flow rate of 3.5 mL/min was used. The eluent consisted of 50% ACN/aq. 20 mM NH_4_HCO_2_ and was adjusted to pH 4.0–4.5 with formic acid.

The ammonium acetate and ammonium formate concentrations stated as aq. 20 mM NH_4_OAc and aq. 20 mM NH_4_HCO_2_, respectively, correspond to the concentration in the aqueous component of an eluent mixture.

The molar activities were determined on the basis of a calibration curve carried out under isocratic HPLC conditions (34% ACN/aq. 20 mM NH_4_OAc; Reprosil-Pur C18-AQ, 250 × 4.6 mm) using chromatograms obtained at 400 nm as an appropriate maximum of UV absorbance (see Fig. S5 in Supplementary Information)^27,28^.

#### Radiochemistry

Manual radiosyntheses. No carrier added [^18^F]fluoride in 1.5 mL water was trapped on a Sep-Pak Accell Plus QMA Carbonate Plus light cartridge (Waters GmbH, Eschborn, Germany). The activity was eluted with 300 µL of an aqueous solution of potassium carbonate (K_2_CO_3_, 1.8 mg, 13 µmol) into a 4 mL V vial prefilled with Kryptofix 2.2.2 (K_2.2.2_, 11 mg, 29 µmol) in 1 mL ACN. The aqueous [^18^F]fluoride was azeotropically dried under vacuum and nitrogen flow within 7–10 min using a single mode microwave (75 W, at 50–60 °C, power cycling mode; Discover PETWave from CEM GmbH Kamp-Lintfort, Germany)^29^. Two aliquots of ACN (2 × 1.0 mL) were added during the drying procedure and the final complex was either dissolved in 500 µL ACN or in 500 µL *tert*-butanol ready for labelling. When [^18^F]TBAF was produced, the aqueous [^18^F]fluoride solution (~300–400 µL) was added to 100–150 µl of tetra-*n*-butylammonium hydrogen carbonate (0.075 M, ABX advanced biochemical compounds GmbH, Radeberg, Germany) dissolved in 1 mL ACN and azeotropically dried as described above. Thereafter, a solution of 1.0 mg of precursor **11** in either 300 µL ACN or 300 µL *tert*-butanol was added, and the ^18^F-labelling was performed at 100 °C. To analyse the reaction mixture and to determine radiochemical yields, samples were taken for radio-HPLC and radio-TLC at different time points (5, 10, 15, and 20 minutes). After cooling to <30 °C, the reaction mixture was diluted with 2.0 mL aqueous NH_4_HCO_2_ (adjusted to pH 4 with formic acid) and 2.0 mL ACN/water (1/1, v/v) and directly applied to an isocratic semi-preparative RP-HPLC for isolation of [^18^F]FACH. The collected radiotracer fraction was diluted with 40 mL water to perform final purification by sorption on a Sep-Pak^®^ C18 light cartridge (Waters, GmbH, Eschborn, Germany) and successive elution with 1.3 mL of ethanol. The ethanolic solution was concentrated under a gentle argon stream at 70 °C to a final volume of 10–50 µL. Afterwards the radiotracer was diluted in isotonic saline to obtain a final product containing 10% of EtOH (v/v)^27^.

Stability and determination of logD value. The stability of [^18^F]FACH was investigated by incubation of small tracer amounts (~5 MBq) at 40 °C in 500 µL *n*-octanol, saline and PBS. After 30 and 60 min, aliquots were taken and analysed by radio-TLC and radio-HPLC. The partition coefficient of [^18^F]FACH was experimentally determined for the *n*-octanol/PBS system by the shake-flask method. Small tracer amounts (~800 kBq) were added to a mixture of 3.0 mL of *n*-octanol and 3.0 mL of PBS. After shaking for 20 min at room temperature, the samples were centrifuged (10,000 rpm, 5 min) and 1 mL aliquots of the organic as well as the aqueous layer were taken and measured in a γ-counter (PerkinElmer Wallac Wizard 1480 Gamma Counter, manufactured by WALLAC, Turku, Finland). Another 1 mL aliquot of the organic layer was mixed with 2.0 mL *n*-octanol and 3.0 mL of PBS and subjected to the same procedure until constant partition coefficient values had been obtained. All measurements were done in quadruplicate^30^.

**Figure 6:**
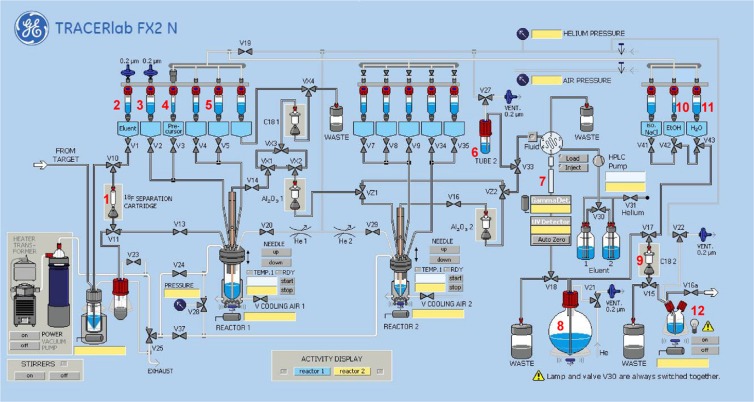
Scheme of the synthesis module TRACERlab FX2 N for the radiosynthesis of [^18^F]FACH. (1) Sep-Pak Accell Plus QMA Carbonate Plus light cartridge, (2) K_2_CO_3_ (1.8 mg in 400 µL water, 100 µl ACN), (3) K_2.2.2._ (11 mg in 1.5 mL ACN), (4) precursor **11** (1.0 mg in 800 µL ACN), (5) 2.0 mL water/ACN (1/1, v/v) and 2.0 mL aqueous NH_4_HCO_2_ (pH 4.0), (6) injection vial, (7) Reprosil-Pur C18-AQ (50% ACN/aq. 20 mM NH_4_HCO_2_, flow 3.5 mL/min), (8) 40 mL water, (9) Sep-Pak^®^ C18 light, (10) 2 mL water, (11) 1.3 mL EtOH, (12) product vial. The scheme of the TRACERlab FX2 N platform was provided by GE Healthcare.

Automated radiosyntheses. Remote controlled radiosynthesis of [^18^F]FACH was performed using a TRACERlab FX2 N synthesis module (GE Healthcare, USA) equipped with a Laboport vacuum pump N810.3FT.18 (KNF Neuburger GmbH, Freiburg, Germany), a BlueShadow UV detector 10D (KNAUER GmbH, Berlin, Germany) and the TRACERlab FX Software. [^18^F]Fluoride (2–6 GBq) was trapped on a Sep-Pak Accell Plus QMA Carbonate Plus light cartridge (Fig. 6, entry **1**) and eluted into the reactor with potassium carbonate (K_2_CO_3_, 1.8 mg, 13 µmol, entry **2**) dissolved in 400 µL water and 100 µL ACN. After addition of Kryptofix 2.2.2. in 1.5 mL ACN (11 mg, 29 µmol, entry **3**), the mixture was azeotropically dried for 5 minutes at 65 °C and 2 minutes at 85 °C. Thereafter, 1.0 mg of the precursor (**11**) dissolved in 800 µL ACN (entry **4**) was added, and the reaction mixture was stirred at 100 °C for 12 min. After cooling, the reaction mixture was diluted with 2.0 mL ACN/water (1/1, v/v) and 2.0 mL aqueous NH_4_HCO_2_ (adjusted to pH 4.0 with formic acid, entry **5**) and transferred into the injection vial (entry **6**). Semi-preparative HPLC was performed using a Reprosil-Pur C18-AQ column (entry **7**). [^18^F]FACH was collected in the dilution vessel (entry **8**) previously loaded with 40 mL H_2_O. Final purification was performed by passing the solution through a Sep-Pak^®^ C18 light cartridge (entry **9**), followed by washing with 2 mL water (entry **10**) and elution of [^18^F]FACH with 1.3 mL EtOH (entry **11**) into the product vial (entry **12**). The ethanolic solution was transferred out of the hot cell and the solvent was reduced under a gentle argon stream at 70 °C to a final volume of 10–50 µL. Afterwards the radiotracer was diluted in isotonic saline to obtain a final product containing 10% of EtOH (v/v)^30^.

**Table 1:** Radiochemical yield in dependence on solvent, ^18^F-fluorination agent and precursor.

Solvent	Precursor	RCY using [^18^F]TBAF	RCY using K[^18^F]F/K_222_
ACN	**11**	54 ± 7%	66 ± 12%
	**11-**Na	9 ± 2%	n.d.
*tert*-BuOH	**11**	10%	n.d.

Selected reaction conditions: 1 mg of precursor, 750–800 µl solvent, 100 °C (thermal heating), 15 minutes; n.d. means not determined.

## Supplementary information

Supplementary Information

## Data Availability

The datasets generated during and/or analysed during the current study are available from the corresponding author on reasonable request.
