# Supplementary material for: One-step radiosynthesis of the MCTs imaging agent [18F]FACH by aliphatic 18F-labelling of a methylsulfonate precursor containing an unprotected carboxylic acid group
Source: Sci Rep. 2019 Dec 11;9:18890. doi: 10.1038/s41598-019-55354-w (PMC6906299; doi:10.1038/s41598-019-55354-w)
Supplement: Supplementary file 1 — Supplementary Information [file 41598_2019_55354_MOESM1_ESM.pdf]

Supplementary Information

to

One-step radiosynthesis of the MCTs imaging agent [^{18}F]FACH: Aliphatic ^{18}F-labelling of a methylsulfonate precursor containing an unprotected carboxylic acid group

Masoud Sadeghzadeh†, Rareş-Petru Moldovan, Rodrigo Teodoro, Peter Brust and Barbara Wenzel†,*

Helmholtz-Zentrum Dresden-Rossendorf, Institute of Radiopharmaceutical Cancer Research,

Permoserstrasse 15, 04318 Leipzig, Germany;

† These authors contributed equally to this work.

***** Author to whom correspondence should be addressed; E-Mail: b.wenzel@hzdr.de;

Content:

Page 1: ^{1}H and ^{13}C NMR spectra of the precursor **11**

Page 2: HPLC chromatograms of the precursor **11** and a representative ^{18}F-labeling reaction mixture of **[^{18}F]FACH**

Page 3: Calibration curve of **FACH** for determination of molar activities

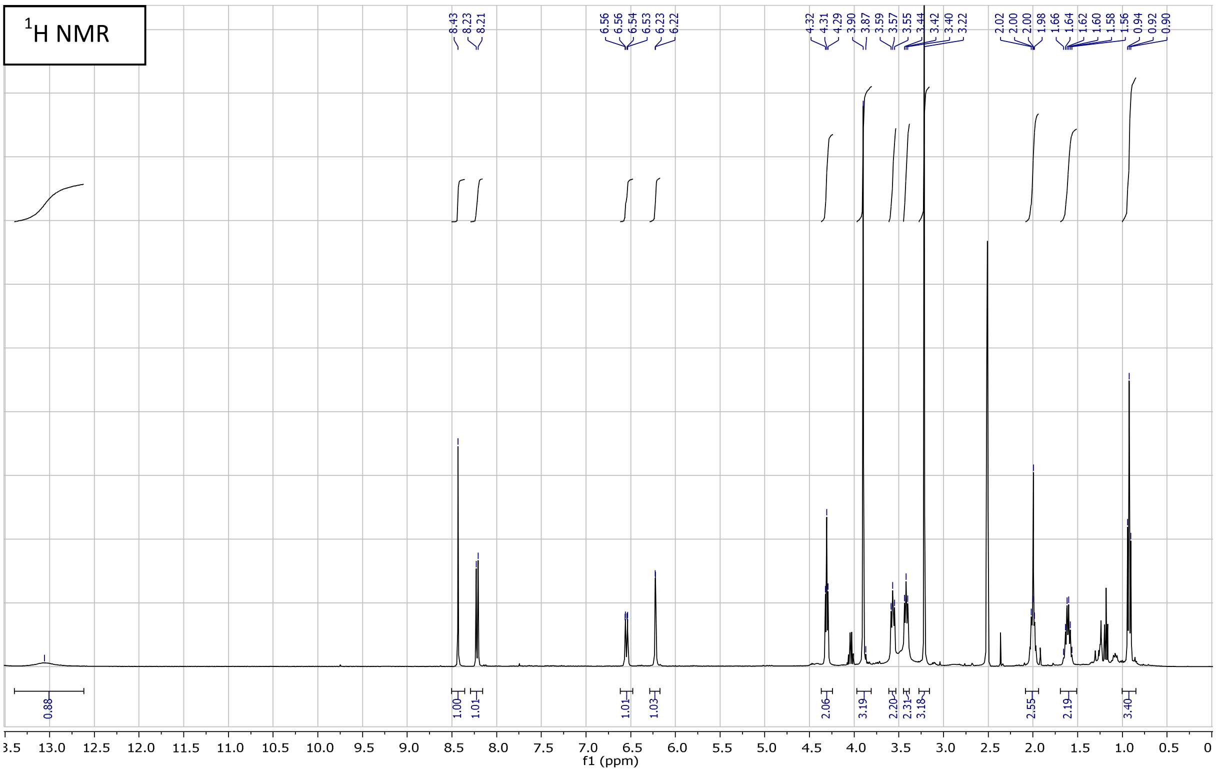

Figure S1: ^{1}H NMR of compound **11** (101 MHz, DMSO-d_6)

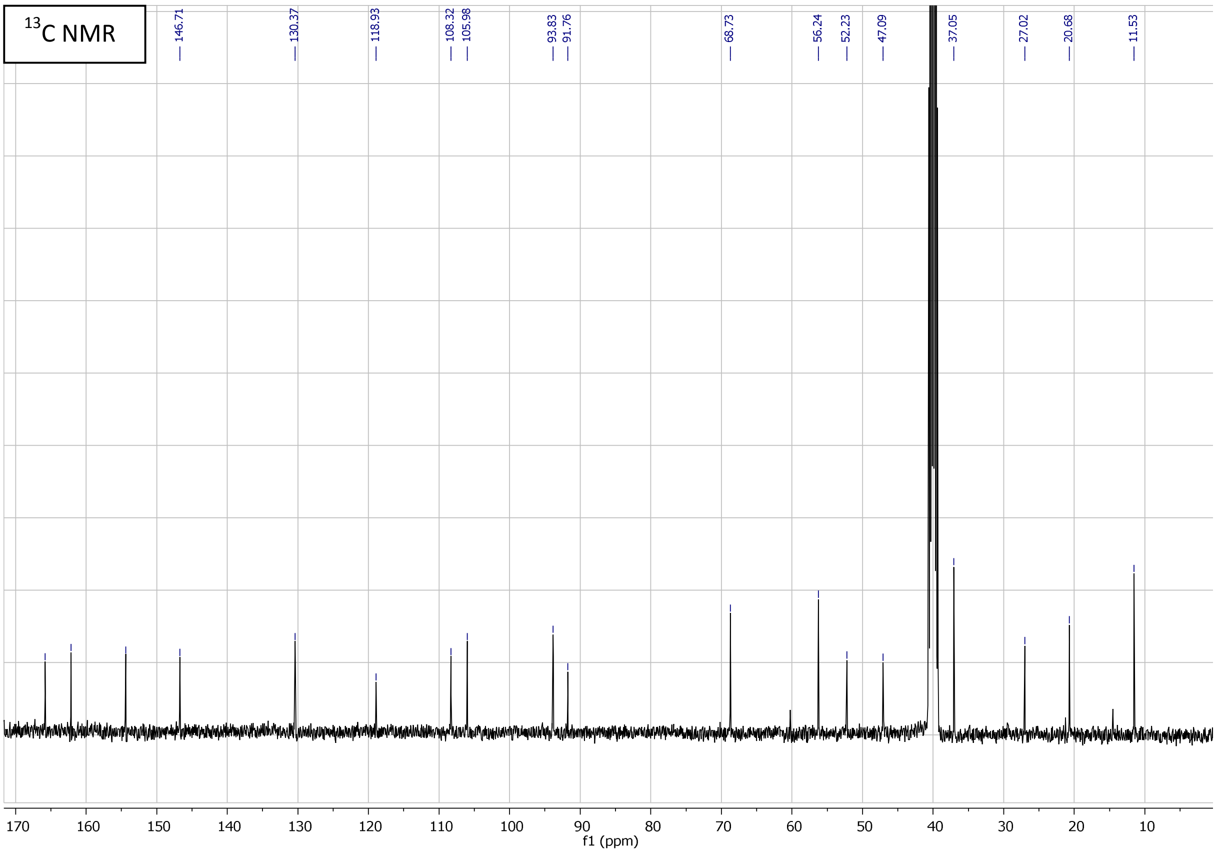

Figure S2: ^{13}C NMR of compound **11** (400 MHz, DMSO-d_6)

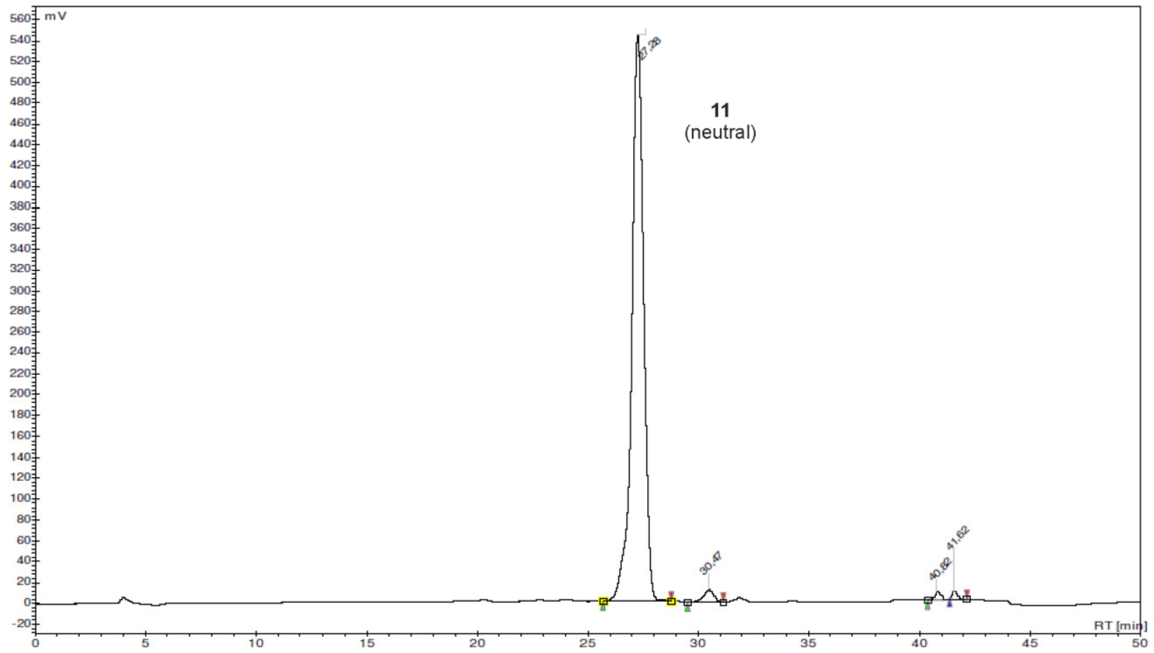

Figure S3: HPLC chromatogram of compound **11** (conditions: Reprosil-Pur C18-AQ, 250 × 4.6 mm, Gradient mode with ACN/aq. 20 mM NH_4OAc, 1.0 mL/min, 254 nm).

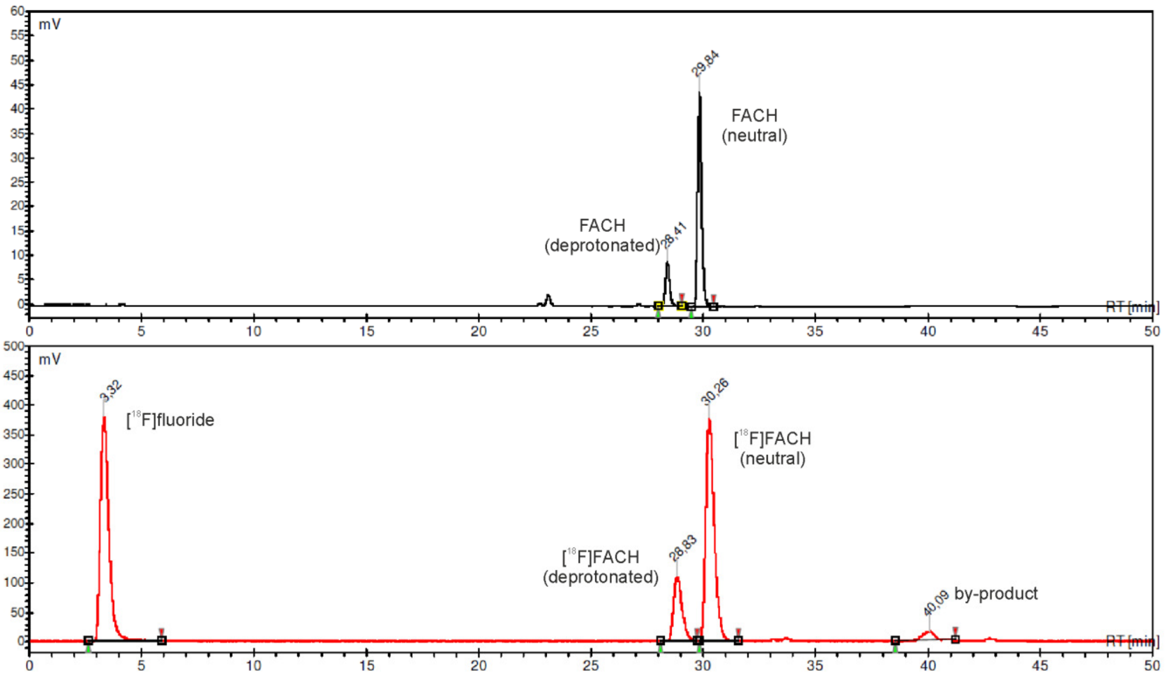

Figure S4: UV- and Radio-HPLC chromatogram of a representative ^{18}F-labelling reaction mixture of **[^{18}F]FACH** spiked with **FACH** (conditions: Reprosil-Pur C18-AQ, 250 × 4.6 mm, Gradient mode with ACN/aq. 20 mM NH_4OAc, 1.0 mL/min).

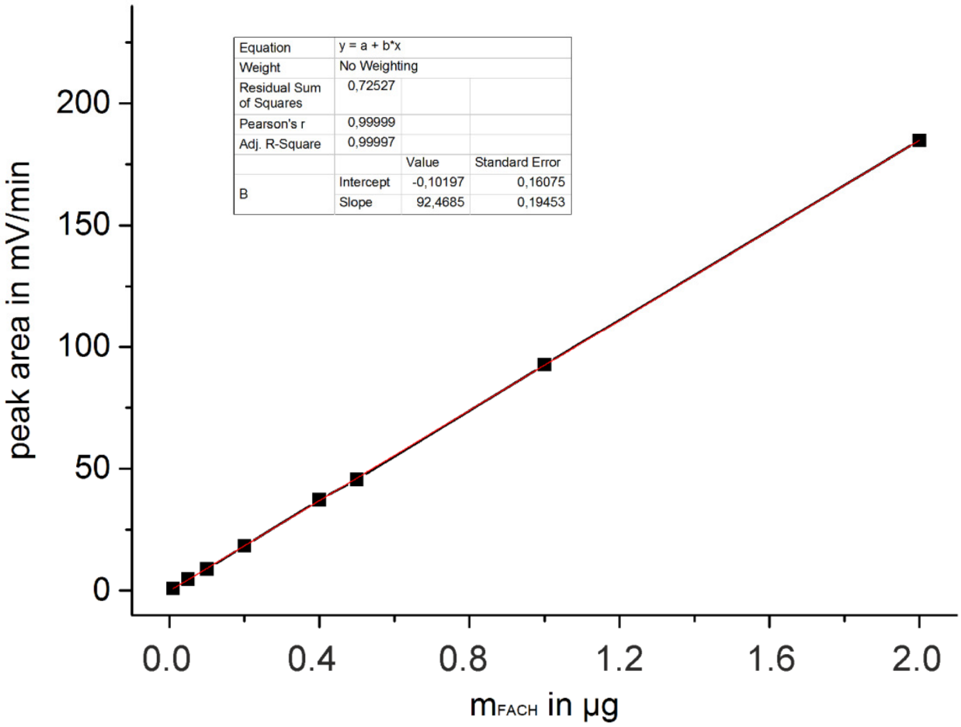

Figure S5: Calibration curve of **FACH** for determination of molar activities (HPLC-conditions: Reprosil-Pur C18-AQ, 250 x 4.6 mm, 34% ACN/20 mM aq. NH_4OAc., 400 nm, 1.0 mL/min).
